# The effectiveness of faculty development programs for training university professors in the health area: a systematic review and meta-analysis

**DOI:** 10.1186/s12909-024-05735-1

**Published:** 2024-07-16

**Authors:** Rosângela Minardi Mitre Cotta, Emily de Souza Ferreira, Fernanda de Aguiar Franco, Gabriel da Costa Souza Barros, José Pedro Toledo Januário, Tiago Ricardo Moreira, Ramón López Martín

**Affiliations:** 1 Department of Health Nutrition, Federal University of Viçosa, Viçosa, MG Brazil; 2 Department of Medicine and Nursing, Federal University of Viçosa, Viçosa, MG Brazil; 3 Facultad de Filosofía y Ciencias de La Educación, Universidad de Valencia, Valencia, VA Spain

**Keywords:** Professionalization in teacher education, Teacher professionalization, Professional development, Professional training

## Abstract

**Background:**

The growing discussion on teacher development focuses on diversified educational skills that promote knowledge and innovation in the teaching, learning and assessment process. With the Covid-19 scenario, this picture of necessary changes has become more evident, demonstrating the need for professional preparation to work in teacher development. The aim of the study was to analyze the effectiveness of teacher development programs for the training of university teachers in the health area, through a systematic review and meta-analysis.

**Methods:**

The systematic review and meta-analysis were carried out according to the Preferred Reporting Items for Systematic Reviews and Meta-Analyses guidelines and involved searching five databases - PubMed-Medline, Education Resource Information Center (ERIC), SCOPUS, Embase and Web of Science. The review included randomized clinical trials and cohort studies that addressed the effectiveness of teaching professionalization in the health area for university professors. The quality of the selected studies was assessed based on the evaluation criteria of the Joanna Briggs Institute tool. The random effects meta-analysis method was used to explain the distribution of effects between the studies, using Stata® software (version 11.0) and publication bias was examined by visual inspection of the graphs and Egger's test.

**Results:**

We included 12 studies in the systematic review and 8 in the meta-analysis. These studies were published between 1984 and 2022 in 14 countries. Significant changes were reported in teachers' behavior to stimulate and encourage students, improvement in the quality of teaching and teaching staff, as well as improvement in skills such as leadership and self-evaluation. Furthermore, the result of the meta-analysis showed that there is evidence of the effectiveness of the positive effects of teacher development programs after their implementation, with this effect being 1.70% and an increase of 4.75 in the effect of these teacher development programs.

**Conclusion:**

Our study shows that development programs have been implemented in different countries and contexts, all of which have proven to be effective in the short, medium and long term. We recommend that future research focus specifically on the different competencies that have been acquired following the implementation of these programs.

## Introduction

Teacher development in higher education is a challenge, especially in developing countries. Although this topic is on university agendas, it is still limited in both its scope and depth of action.

In this context, educators need to have diversified educational skills and play the role of knowledge promoters, placing the student as the main agent in this process [1–3].

Therefore, in order to keep up with changes and guarantee quality teaching, teacher development programs have been increasingly valued as a strategy used to prepare teachers for their jobs. The literature describes advantages for institutions that rely on this type of program, such as greater motivation, improved skills and a better ability to manage change [4]. In addition, these types of faculty development programs have been considered a relevant autonomous educational pedagogy to promote professional skills and knowledge [5].

The Covid-19 pandemic has highlighted a reality in which many professionals were unprepared, i.e. with the advancement of science and technology in the health area, it is necessary to train more critical professionals, with a view to individualizing care, as well as backed up by the best evidence [6, 7].

Recently, after the Covid-19 pandemic, teacher professionalization programs have gained even more importance [6, 7], studies point to a great difficulty in identifying innovative and lasting teacher development programs, in order to maintain a longitudinal evaluation of the participants' progress and most of the work is centered in Europe and the United States of America.

In this sense, it is essential to analyze training from teacher development programs, as a knowledge-forming agent, with scientific rigor, in order to evaluate the effectiveness of different types of programs. The scientific literature shows that it is necessary to incorporate teacher development programs into institutions in order to improve the academic performance of teaching staff and consequently benefit students, and that there has been a significant increase in these programs. However, few studies have focused on the programs that have been created and implemented for the training of university teachers in the health area and which focus on concrete changes in teaching practice, based on the skills that have been acquired and incorporated.

This study stands out for having been carried out by means of a systematic review with meta-analysis on the subject of teacher professionalization with a focus on the health area and the programs implemented that have brought about concrete changes in teaching practice. The aim of the study was to analyze the effectiveness of teacher development programs for the training of university teachers in the health area, by means of a systematic review and meta-analysis.

## Methods

### Study protocol and registration

This systematic review and meta-analysis were conducted in accordance with the Preferred Reporting Items for Systematic Reviews and Meta Analyses (PRISMA) [8] and registered with the International Prospective Register of Systematic Reviews (PROSPERO) under protocol number CRD42023427829.

### Search strategy

A preliminary search was carried out with the help of a senior librarian to specify the keywords and optimize the search strategy. The databases were searched using terms from the Medical Subject Heading (MeSH), Emtree and Thesaurus. The following descriptors were used: "Teacher Training", "Teacher Education", "Faculty", "Health Personnel", "Health care personnel", "Faculty development" "Effectiveness", "Efficacy" and "Efficiency". To take into account the perspective of teacher development programs and their effectiveness, the search terms were used in the following combination: (teacher training OR teacher education) AND (faculty OR faculty development) AND (health personnel OR health care personnel) AND (effectiveness OR efficacy OR efficiency).

### Data sources

The data sources for this systematic review were the electronic databases: Medical Literature Analysis and Retrieval System Online (PubMed-Medline), Education Resource Information Center (ERIC), Excerpta Medica dataBASE (EMBASE), SCOPUS and Web of Science. The last search was carried out in July 2023 to identify eligible studies and no date restriction filter was used.

### Study selection

The review included randomized clinical trials and cohort studies that addressed the effectiveness of teaching professionalization in the health area for university professors. Studies were included if they explicitly related to the professionalization of university teachers in health areas. We excluded non-original studies and studies from which it was not possible to extract relevant data for the analysis, such as letters, editorials, conference proceedings, comments, reports, study protocols, pilot studies, abstracts and reviews. In addition, studies that did not address teacher professionalization in non-health areas, as well as teacher professionalization in non-university teachers, were excluded. There were no date, location or language restrictions.

The title, abstract and full text were screened independently by three reviewers (FAF; GB; JPJ), and disagreements were discussed by another reviewer (ESF). Each of the three reviewers selected the studies for possible inclusion on the basis of the title and the content of the abstract. Studies considered to meet the inclusion criteria were analyzed in the full-text review.

The articles were first selected based on their titles. After reading the abstract, those that did not fit the research were excluded, taking into account the exclusion criteria. If the abstracts were not available, the full-text articles were retrieved for evaluation. After this selection of articles, all the selected articles were read in full to examine compliance with the inclusion criteria. Any disagreement in the assessment of the articles was resolved through discussion within the review team.

### Quality of the studies

The quality of the selected studies was assessed based on the evaluation criteria of the Joanna Briggs Institute tool [9] specific to cohort studies and randomized clinical trials. The results were measured in percentages for each item on the checklist: 1 point for "YES", 0.5 points for "unclear" and 0 for "NO". Good quality studies were those that scored above 75% [10].

### Data extraction

Rayyan software was used to analyze the titles and abstracts. Three authors (FAF; GB; JPJ) extracted all the data and one reviewer (ESF) analyzed the data for accuracy. The following data was collected: title of the article, authors, country, duration of the study, year of publication of the article; type of study; name of the teacher professionalization program; number of participants; sample characteristics; program approach; program data and main results achieved.

Quantitative data was also extracted for initial (baseline) and final values in both the control and intervention groups for most studies, otherwise only final values were extracted. When follow-up values were missing (e.g. standard deviation), the final intervention values were selected to estimate their effects.

We created a Microsoft Excel spreadsheet to extract all the information mentioned above.

### Data synthesis and analysis

The meta-analysis was carried out using fixed and random models (where necessary) and Weighted Mean Difference (WMD) to calculate the effectiveness of teacher professionalization programs. We calculated the Standard Deviation (SD) of some studies using data imputation. Heterogeneity was assessed using the chi-squared test (χ2) with a significance level of 90% (p < 0.10), and its magnitude was determined by the I-squared (I2) [11]. Thus, heterogeneity was classified as low, moderate or high when the I-squared values were above 25, 50 and 75%, respectively. The results were summarized by means of a meta-analysis of the means and relative risk of the pre-test and post-test groups, with the respective SD.

The meta-analysis was carried out using Stata® software (version 11.0) and publication bias was examined by visual inspection of funnel plots and the Egger test.

The risk of bias was also analyzed using RevMan (version 5.4). The statistical significance of the overall effect size of implementing faculty development programs was determined by the 95% confidence interval (CI).

## Results

### Studies identified and included

**Fig. 1:**
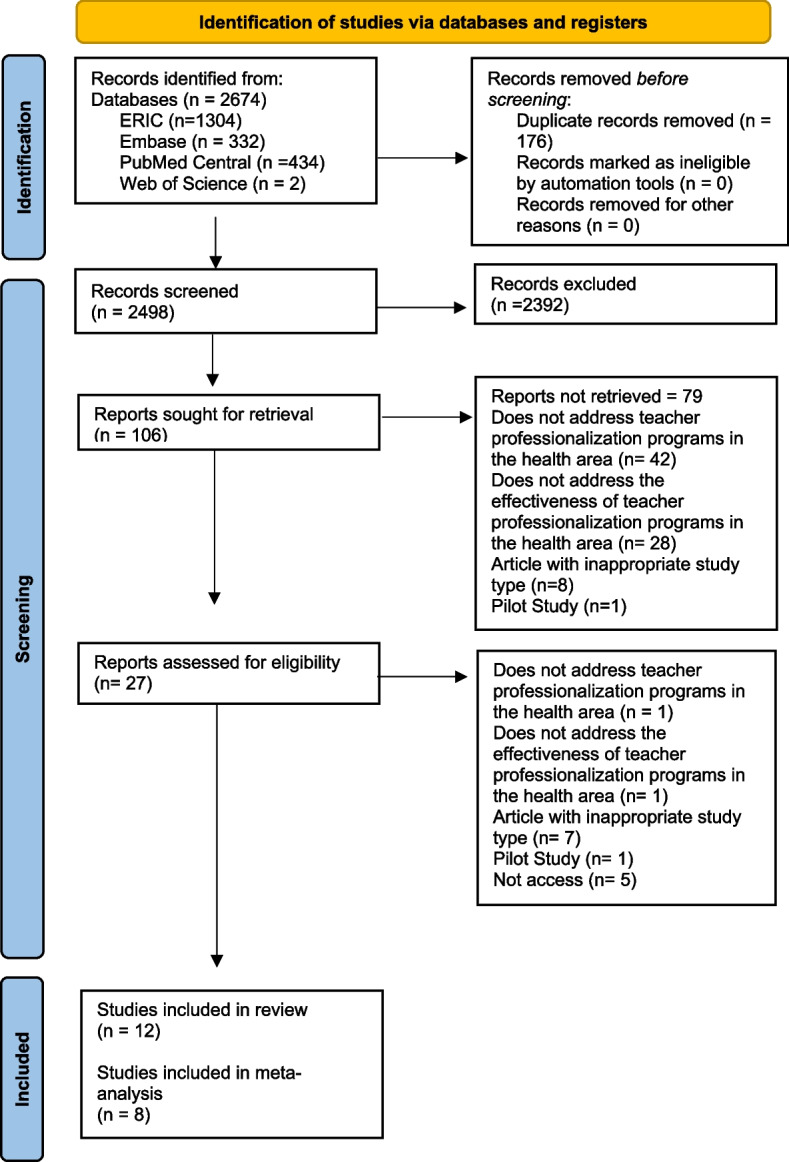
Flowchart of preferred reporting items for systematic reviews and meta-analyses (PRISMA) study selection

We searched four databases and obtained 2674 articles. The first step was to exclude duplicate articles using Rayyan software, which left us with 2498 articles. In the second stage, studies were excluded by screening titles and abstracts, which left us with 106 articles. In the third stage, the studies were excluded by carefully screening the full text of the articles. Finally, we included 12 articles in the systematic review, 8 of which had all the data required for inclusion in the meta-analysis. The retrieval and selection process are shown in Fig. 1.

For articles to be included in the meta-analysis, it is necessary that they have quantitative data relating to the aspects they propose to evaluate. This data can be mean or median values and their respective standard deviation or interquartile range. The four articles that were not included in the meta-analysis did not have this data in its entirety, which made it impossible for us to use them for statistical analysis.

### Characteristics of the included studies

There were 412 participants in the 12 studies, which were from 14 countries (Israel [1], United States of America [7], Switzerland [1], United Kingdom [1], Korea [1], India [1]). The time of publication ranged from 1984 to 2022. The average sample size was 34 individuals. In terms of study design, all were cohort studies. As for the profile of the study participants, faculty members, hospital clinical supervisors and doctors and nurse educators stand out.

As for the results, significant changes were seen in teachers' behavior to stimulate and encourage students, improvement in the quality of teaching and teaching staff, improvement in skills such as leadership and self-assessment by teachers, improvement in the level of confidence in teaching with the recognition of new ways of teaching and the identification of a new assessment culture, in a more flexible way and using existing infrastructure. Although each study included in the systematic review addresses a different program and evaluates different aspects, we can highlight that they have in common, the study design (longitudinal) and in all studies, after the end of the implemented programs, the participants (teachers and students) perceived positive changes and reported satisfaction with the results achieved, including the development of new skills, especially those linked to clinical reasoning. All these results are detailed in Chart 1, Additional file 1.

### Meta-analysis

Of the 12 articles included in the systematic review, 8 compared the effectiveness of teacher development programs for the training of university teachers in the area of health, before and after their implementation. A total of 332 people were included. All these results are detailed in Chart 2, Additional file 2.

**Fig. 2:**
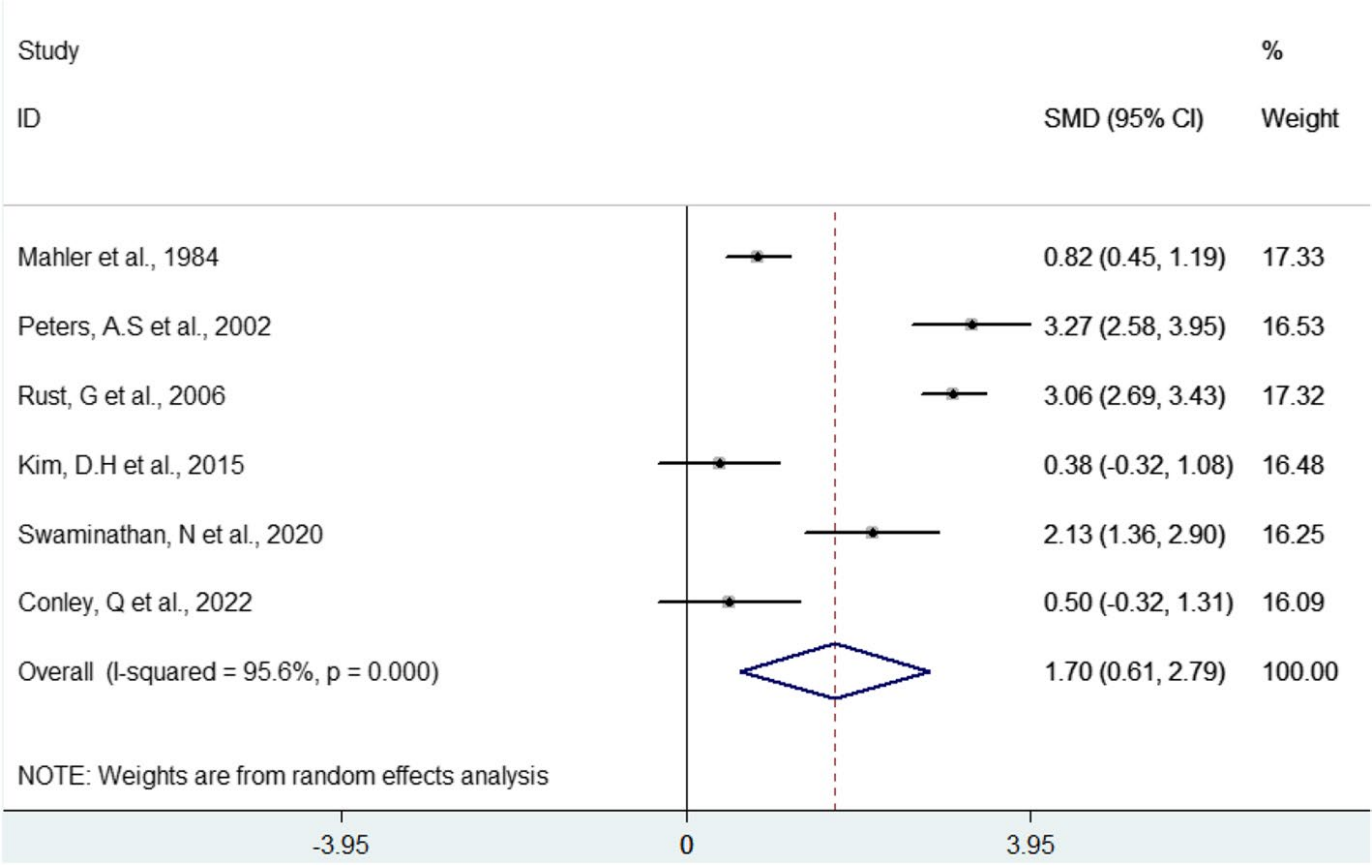
Forest plot of the effects of implementing teacher development programs, based on average scores

**Fig. 3:**
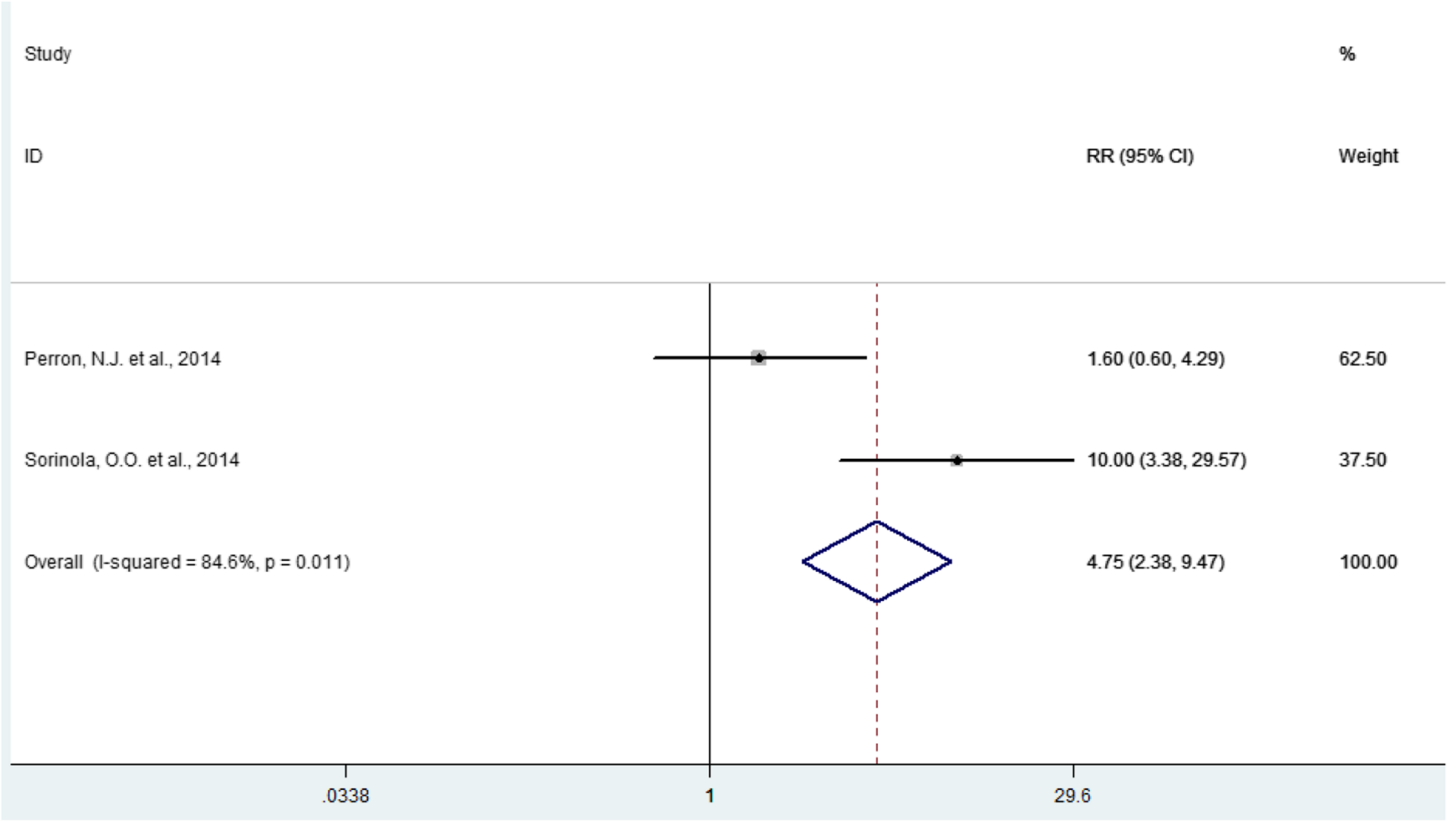
Forest plot of the effects of implementing teacher development programs, based on relative risk

As shown in Figs. 2 and 3, the eligible articles have a weighted mean value of improvement or worsening after the implementation of the programs and a confidence interval and weight that varies according to the sample size of the article. Both figures show that most of the studies indicate that after the implementation of the programs, there was a significant difference when compared to the scenario before any teacher development program was implemented.

This indicates that the average score was higher after the intervention, which indicates that there is a positive effect and effectiveness of the teacher development programs after their implementation.

The overall effect size and respective positive effect was 1.70% (CI 0.61—2.79) (*p* = 0.000) (Fig. 2). Also, after the intervention, there was an increase of 4.75 (CI 2.38—9.47) in the effect of the teacher development programs in the scenarios in which they were applied (Fig. 3). Analyzing the results of the articles included in the meta-analysis, this result means that the teacher development programs analyzed made the participants learn and were able to implement changes in teaching practice. These changes include knowledge, skills and attitudes that provide practical improvement for educators through learning objectives, instructional strategies, communication, motivation and feedback.

There was a high degree of heterogeneity between the studies in the two meta-analyses carried out (95.6%; *p* < 0.001; 84.6%; *p* = 0.011), which indicates great variation in the results of the studies, which can be explained by the different types of programs that were implemented in different teaching settings.

### Risk of bias

**Fig. 4:**
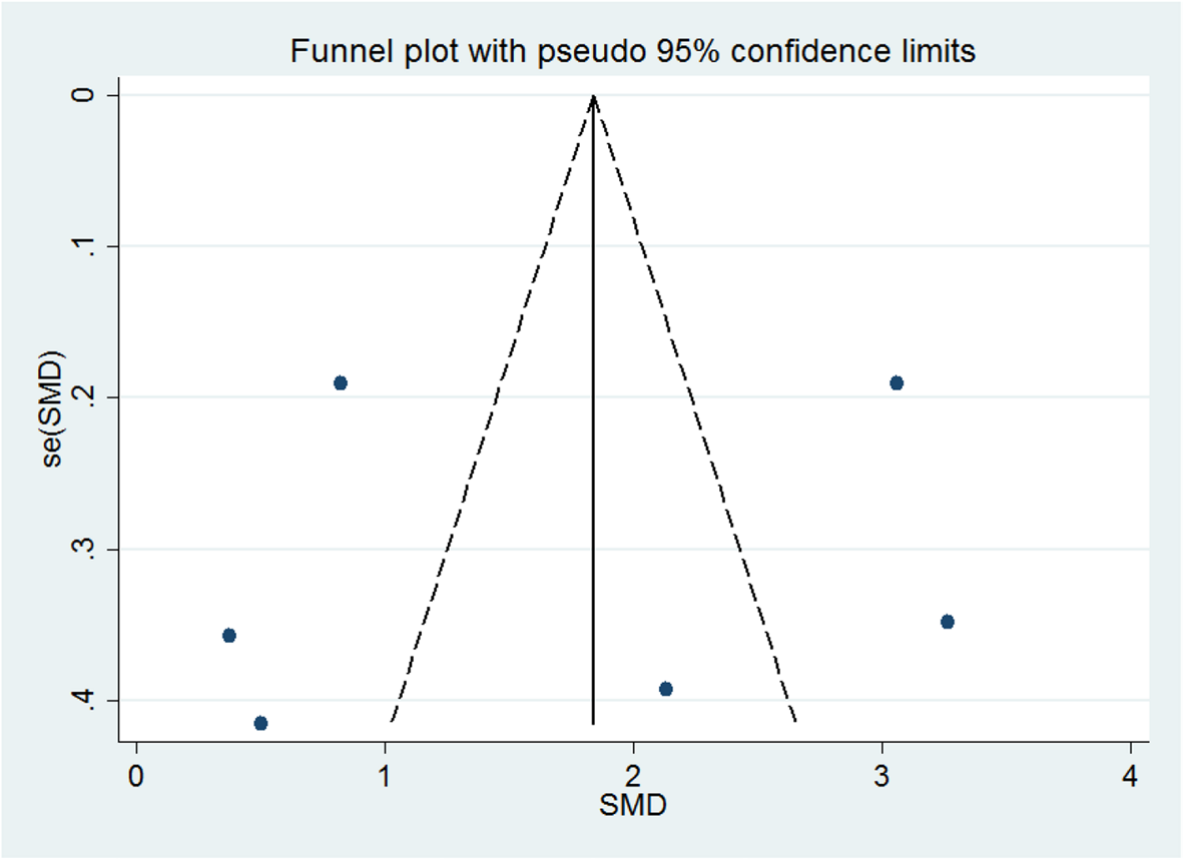
Funnel plot. WMD, Funnel plot with 95% confidence limits, based on mean scores

**Fig. 5:**
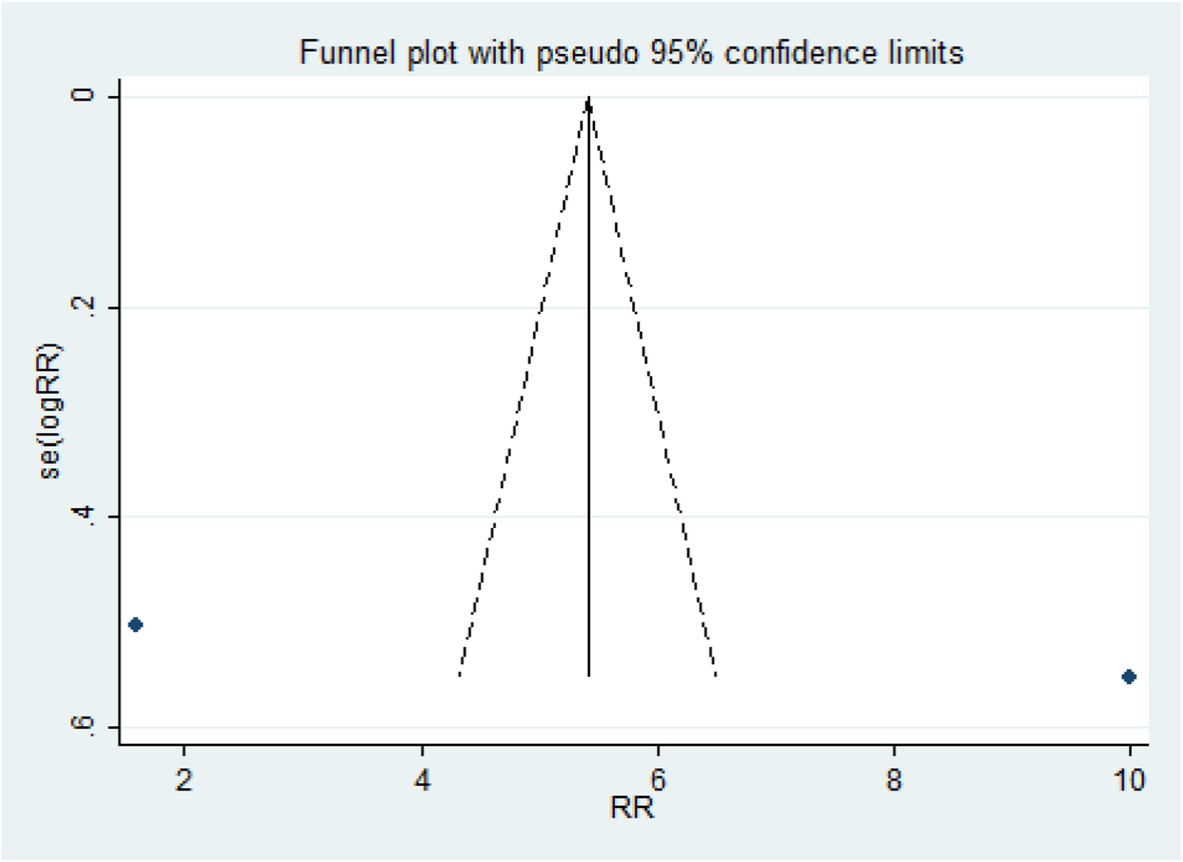
Funnel plot. WMD, Funnel plot with 95% confidence limits, based on relative risk

Figures 4 and 5 show the results of the "meta funnel", a test carried out to investigate the risk of publication bias. In our analysis, in both figures, only one article fell within the boundaries of the funnel, which indicates a risk of bias that can be justified by the high heterogeneity reported earlier. However, Egger's test indicated non-significant results (p = 0.759), which reinforces that the bias found is not represented by the quality of the data analyzed, but rather by the variety of programs that were obtained from the studies analyzed.

**Fig. 6:**
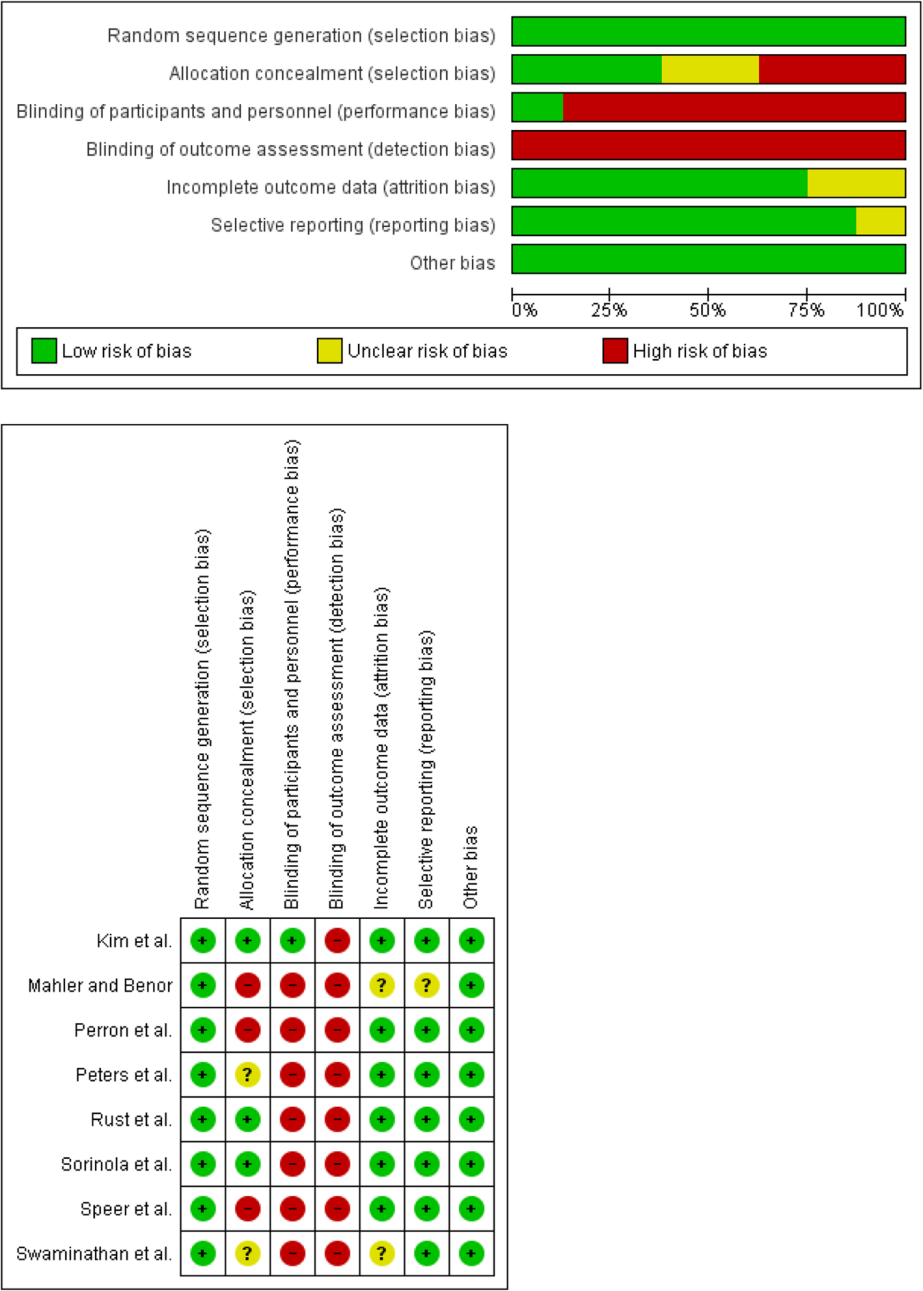
Summary of the risk of bias for each study included in the meta-analysis

We also calculated the specific risk of bias, which is shown in Fig. 6. In summary, no article fully met the quality criteria, however, none presented a high risk in all the criteria evaluated, so all were included in the meta-analysis carried out. No study blinded the results achieved and all of them used random sequence generation to define the sample, so selection bias was low.

With regard to reporting bias, most of the studies (7/8) had a low risk of bias, as they clearly expressed the characteristics of the participants in their respective studies. Most of the articles (6/8) presented results and follow-ups, so the risk of attrition bias was low.

Non-blinding of the participants (performance bias) and of the results (detection bias) presented a high risk of bias. This can be explained by the objective proposed in this systematic review and meta-analysis and, consequently, in the methodology that was conducted in each article analyzed, since all the participants took an active part in the whole process of teaching and learning the methods and programs implemented, so the results they achieved throughout the process were perceived by the participants, and it was impossible to blind them to these results.

### Quality assessment

Of the 12 articles included in the systematic review, 10 scored between 50 and 75% in the quality assessment, 1 article scored less than 50% and 1 article scored above 75% in the assessment criteria of the Joanna Briggs Institute forms (Table 1). No study was of low quality.

**Table 1:** Assessment of the quality of the studies included in the systematic review

**Id**	**Title of the article**	**Score/number of questions**	**Quality (%)**
**1**	Short- and Long-Term Effects of a Teacher-Training Workshop in Medical School	6,5/11	59,09%
**2**	Evaluation of a faculty development program in managing care	6/8	75,00%
**3**	The morehouse faculty development program: Evolving methods and 10-year outcome	7/11	63,64%
**4**	Long-term follow-up of a dental faculty development program	8/11	72,70%
**5**	Impact of a faculty development programme for teaching communication skills on participants’ practice	7/11	63,64%
**6**	Faculty Development for Educators: A Realist Evaluation	5/11	45,45%
**7**	Evaluation of an international faculty development program for developing countries in Asia: the Seoul Intensive Course for Medical Educators	8/11	72,73%
**8**	Faculty Development for Fostering Clinical Reasoning Skills in Early Medical Students Using a Modified Bayesian Approach	4/8	50,00%
**9**	Implementing Systematic Faculty Development to Support an EPA-Based Program of Assessment: Strategies, Outcomes, and Lessons Learned	5,5/8	68,75%
**10**	Evaluating the effectiveness of an online faculty development programme for nurse educators about remote teaching during COVID-19	7/9	77,77%
**11**	A mixed-methods study of the effectiveness and perceptions of a course design institute for health science educators	7/11	63,64%
**12**	Peer observation of teaching: A feasible and effective method of physician faculty development	8/11	72,73%

## Discussion

The results found showed that most of the study participants were faculty members, hospital clinical supervisors and physicians and nurse educators, all of whom wanted to improve their teaching development. In all the studies included in the systematic review, after the completion of the programs that were implemented, the participants (faculty and students) perceived positive changes and reported satisfaction with the results achieved, among them the development of new skills, especially those linked to clinical reasoning.

In addition, the meta-analysis conducted in our article showed that there is a positive effect and effectiveness of faculty development programs after their implementation. This positive effect was 1.70% (CI 0.61—2.79). Furthermore, after the intervention, there was an increase of 4.75 (CI 2.38—9.47) in the effect of the teacher development programs in the scenarios in which they were applied.

In this context, development programs are designed to prepare institutions and faculty members in areas such as teaching, research, administration and career management [12]. As far as medicine is concerned, data shows that faculty development programs began in the late 1970s, driven by the demand for more innovative teaching. In addition to a continued focus on teaching, faculty development has been shown to encompass other faculty functions, such as organizational and leadership development [12, 13]. Widespread investment in faculty development is based on the belief that it improves the effectiveness of teaching and learning, although evidence of long-term impact is limited.

Thus, the articles analyzed highlighted significant changes in the behavior of teachers, with greater emphasis on stimulating and encouraging students, improving the quality of the teaching staff and consequently the teaching given to future medical professionals. This is in line with what is expected from the training of health professionals in the twenty-first century [3]. In this sense, health professionals in the twenty-first century are expected to be able to keep up to date with the latest information and communication technologies, as well as scientific technologies, in order to integrate more efficiently into their field of work. At the same time, it is necessary to train professionals who are critical and foster analytical thinking, prepared to learn by researching reliable sources with autonomy and responsibility, with a view to making the best use of all scientific and technological advances, without, for example, leading to unnecessary examinations or procedures [14, 15].

Improved competencies such as leadership and self-assessment on the part of teachers were also observed as a result, which is highlighted in the study by Bray et al. (2020) in which the creation of a leadership group with educational expertise, political and strategic capacity was sought, resulting in greater effectiveness of teacher development efforts in the institutionalization of the assessment program through the training of skills such as direct observation, feedback and consistent use of standards-based criteria [14]. In addition, the studies previously analyzed found an improvement in the level of confidence in teaching, with the recognition of new ways of teaching. In this context, the use of simulations to improve non-technical skills such as communication, situational awareness and teamwork has been shown to be effective in improving teaching [15–17].

In addition, other strategies have been pointed out, mainly driven in recent years by the Covid-19 pandemic and the discouragement of face-to-face meetings as a health measure [18, 19], the use of emerging technologies such as metaverse [18], investigating the effects with students, as well as the use of google classroom, bringing greater flexibility and encouraging active learning. In this way, the development of online tools in teacher professionalization programs is increasingly gaining ground in research [14, 18].

However, for faculty development programs to be disseminated in universities, some challenges need to be overcome, such as the lack of institutional incentive [6, 18], the lack of training in faculty professionalization, the need for resources [16, 17] and the lack of time [17, 18]. In this context, when health professionals who become educators in higher education institutions are not prepared for the transition from their clinical skills to teaching, it can negatively affect the students' experience and also lead to job dissatisfaction among new teachers, which can lead to frustration and burnout [6, 12, 17, 20]. It is necessary to question the voluntary nature of faculty development and its impact on institutional culture and the values attributed to teaching and learning [21], because although health professionals are experts in what they teach, they generally have little training in how to teach [12].

In this way, teacher development programs have proved to be fundamental, since continuous updating with regard to the teaching–learning process proves to be effective for individuals who work in the health area, for example, teachers, students and professionals, also making it possible to identify a new culture of evaluation, in a more flexible way and using the infrastructure that already exists in search of the formation of critical, reflective professionals, with the capacity to self-correct and act with co-responsibility [14].

Steiner et al. [21] carried out a systematic review comparing teacher development methods and analyzing which characteristics contribute to changes in teacher performance. The results showed that it is necessary to implement long-term teacher development programs, allowing the accumulation of technical and practical knowledge over time and seeking close collaboration with research colleagues. In addition, the authors pointed out that it is important to implement the development of methods to assess the impact of teacher evaluation in institutions in a systematic way, fostering interdisciplinary collaboration [21].

This study may have some limitations. The first is that most of the studies were carried out in the USA, which means that they are not geographically comprehensive. Secondly, we were unable to include all the articles included in the Systematic Review in the Meta-analysis, due to the absence of crucial data for this analysis. The third is that most of the articles included do not consistently provide a joint base of academic skills developed with the implementation of faculty development programs, and those that did address this aspect did not exceed 1 year of follow-up.

## Conclusions

A total of 12 articles were included in the systematic review of which 8 were eligible for the meta-analysis. All articles focused on the effectiveness of faculty development programs for the training of university professors in the health field.

Based on our findings, we can conclude that there is no specific type of program that is more or less effective, as they all generated positive impacts. Furthermore, the definition of a more or less effective program depends on the way it is implemented and evaluated within the context of teacher development. However, based on the results found in the 12 studies analyzed, we can say that a good teacher development program must have a realistic approach, which considers the pre and posttest, with a minimum duration of 3 months (preferably done in a course format) and that is designed considering an evaluation through continuous feedback, in order to evaluate whether, in fact, the implemented program is helping to develop skills in both the teacher and the student.

Our results showed too that after the implementation of the teacher development programs, all participants changed their teaching practice positively, incorporating the results achieved into their practice, especially those related to the development of new competencies. Furthermore, the meta-analysis reinforced these positive changes in the scenarios in which they were applied.

In summary, our study shows that development programs have been implemented in different countries and contexts, all of which have proven to be effective in the short, medium and long term. We recommend that future research focus specifically on (1) the different competencies that have been acquired following the implementation of these programs, as not all the articles analyzed focused on this aspect; (2) additional investigations to explore the impact of programs in the long term (larger cohort) and with more participants; (3) incorporation of additional pre/post testing instruments that can quantify and specify the changes made; (4) the incorporation of different points of view from the parties involved, which includes the student and their learning experiences with the new program that was implemented.

## Data Availability

Data is provided within the manuscript or supplementary information files.

## Abbreviations

PRISMA: Preferred Reporting Items for Systematic Reviews and Meta Analyses
PROSPERO: International Prospective Register of Systematic Reviews
MESH: Medical Subject Heading
ERIC: Education Resource Information Center
WMD: Weighted Mean Difference
SD: Standard Deviation
CI: Confidence interval
